# Team interactions in robotic-assisted surgery: a scoping review

**DOI:** 10.1007/s11701-025-02850-z

**Published:** 2025-10-21

**Authors:** Kyi Phyu Nyein, Kyle M. Canady, Joshua A. Duron, Jessica L. Wildman

**Affiliations:** 1 Department of Psychology, University of Zurich, Binzmüehlestrasse 14, Box 1, 8050 Zurich, Switzerland; 2 School of Psychology, Florida Institute of Technology, Melbourne, Florida USA

**Keywords:** Robotic-assisted surgery, Teamwork, Communication, Non-technical skills, Flow disruptions, Surgical education

## Abstract

Robotic-assisted surgery (RAS) significantly alters the operating theater team’s spatial configuration, necessitating different communication strategies that subsequently affect team dynamics. Despite the growing prevalence of RAS, inconsistencies remain regarding the conceptualization and measurement of team interactions specifically in RAS in the existing literature. This scoping review aims to provide an overview of the empirical research on team interactions within RAS, identify research gaps, and propose future directions. We conducted the scoping review following updated methodological guidelines and Preferred Reporting Items for Systematic reviews and Meta-Analyses extension for Scoping Reviews (PRISMA-ScR) reporting standards. Searches of PsycINFO, CINAHL, PubMed, Cochrane, Embase, MEDLINE, Web of Science, and Scopus databases were performed, including empirical articles published between January 2010 and October 2023 focusing on team interactions in RAS. Fifty-four empirical articles were included for analysis. Findings highlighted four major themes: teamwork and communication, non-technical skills, flow disruptions, and teaching/surgical education. Most included studies used quantitative methods and observational data. Communication was frequently studied but inconsistently defined and measured, whereas non-technical skills were more consistently defined and measured. Flow disruptions significantly impacted surgical outcomes, and studies emphasized the need for education on verbal communication strategies in RAS. This review illustrates the inconsistencies in defining and measuring team-related concepts in the literature, highlighting the need for standardized terminologies and measures. Future research should explore mediators and moderators in team interactions in RAS and integrate non-technical skills into training curricula. Such advancements could enhance surgical training, team effectiveness, patient safety, and outcomes associated with RAS.

**Supplementary Information:**

The online version contains supplementary material available at 10.1007/s11701-025-02850-z.

Robotic-assisted surgery (RAS) has become increasingly common in recent years [1] as it boasts advantages over traditional open or laparoscopic surgery, such as improved surgeon’s vision, dexterity, precision, and ergonomic position as well as patient outcomes (e.g., reduced length of incision and blood loss, faster recovery with less pain, and reduced hospital stay) [2–5]. Despite the growing use of RAS for various surgical procedures, challenges have been noted regarding intraoperative communications and interactions among team members [6]. RAS introduces a change in the physical configuration of the operating theater team and patient such that the surgeon sits at a remote console, physically removed from the patient, while the rest of the team remains at the bedside in the sterile field [3, 4]. The implication of this change in configuration is that the surgeon has reduced vision on the operating field with a closed console, and the team members’ view can also be blocked by the robotic equipment [3, 4]. This dispersed configuration heightens the need for the team members to verbalize their instructions and actions in an explicit and deliberate manner [6] that does not rely on nonverbal cues that may be missed when using the closed console. In addition, as the surgeon is physically separated from the rest of the team, the team must address events at the bedside and communicate them to the surgeon to keep the surgeon informed [6]. Therefore, effective team interactions have been particularly highlighted as one of the most important factors affecting patient safety in RAS settings [6, 7].

Previous reviews have examined team interactions in RAS through a variety of perspectives, such as workflow efficiency [4], skill assessment [8, 9], RAS system integration through human factors principles [6, 10], factors influencing patient safety [7], and RAS impacts on team performance and decision making [3, 8]. While there is value in studying team interactions in RAS through respective lenses, this scattered literature results in a lack of understanding of what all of these perspectives mean holistically. In other words, there is a lack of integrated knowledge on how team interactions are studied and understood in the literature. Moreover, there is a lack of a standardized approach to studying team interactions in RAS such that various concepts related to team interactions are studied using the same label, or the same concept using different labels. As a result, it creates confusion regarding what is known and what is still not known about the topic.

Therefore, the goal of this scoping review is to examine how team-related concepts have been conceptualized and studied in RAS. Its objectives are threefold. First, it will provide an integrated overview of how empirical research using quantitative, qualitative, and mixed methods has been conducted on team interactions in RAS. Second, it will discuss terminologies and definitions used to capture team-related concepts in RAS. Third, it will present gaps in the literature and identify future research and needs to further advance the knowledge regarding the conceptualization, measurement, role, and practice of team interactions in RAS.

## Method

We followed Peters et al.’s updated methodological guidelines for conducting scoping reviews [11] and used Preferred Reporting Items for Systematic reviews and Meta-Analyses extension for Scoping Reviews (PRISMA-ScR) for reporting the results [12]. Please see Supplemental Material 1 for the PRISMA-ScR checklist. This scoping review is part of the larger study to develop an observational framework of team communication in RAS. The study protocol, including steps for this scoping review, has been published in JMIR Research Protocol [13]. We conducted our scoping review in the following stages: (1) developing inclusion and exclusion criteria, (2) developing search strategy, (3) screening and selecting abstracts and full texts, and (4) extracting and analyzing the data.

### Developing inclusion and exclusion criteria

As the topic of this scoping review is interdisciplinary, we included studies published in any discipline and country. Based on this scope, we developed inclusion and exclusion criteria (Table 1).

**Table 1:** Inclusion and exclusion criteria

	Inclusion criteria	Exclusion criteria
Publication Date	Between January 2010 and October 2023	Before 2010
Target Population	Healthcare professionals and students	Non-healthcare professionals and students
Target Context	Hospital, medical center, operating theater, robotic teaching and learning environment, simulated environment	Community (e.g., nursing home) where there is no surgery performed
Type of Surgery	Robotic surgery, comparison of robotic surgery to other types of surgery	Laparoscopic surgery, open surgery
Sources of Evidence	Peer-reviewed articles, conference proceedings or papers, theses, and dissertations	Books, periodicals, magazines, policy documents, websites
Language	English	Non-English
Type of Evidence	Empirical (quantitative, qualitative, mixed methods)	Non-empirical (theoretical, conceptual, review)

### Developing search strategy

Following suggestions of a medical librarian, we conducted a literature search in January and February 2024 in the following databases: PsycINFO, CINAHL, PubMed, Cochrane, Embase, MEDLINE, Web of Science, and Scopus. Please see Supplemental Material 2 for the search terms and search query we used in PubMed as an example.

### Screening and selecting abstracts and full texts

The research team included doctorate- and master-level industrial and organizational psychologists with expertise in team dynamics and human factors. KPN and KMC independently conducted abstract and title screening as well as full-text screening on Covidence, and JLW resolved any disagreements. Throughout the process, the team met regularly to discuss any discrepancies and revise the inclusion and exclusion criteria as necessary until an agreement was reached.

### Extracting and analyzing the data

We developed a data charting form in Microsoft Excel based on this review’s aim and objectives. The entire team (KPN, KMC, JAD, and JLW) first extracted data from five included articles independently using the charting form. Then, they met to resolve any disagreements and revise the charting form iteratively. The charting form recorded key information about the articles including publication year, journal, research theme, surgical specialty of the sample, RAS system used, study design, study setting, participant type, focal constructs, role of focal constructs (e.g., antecedent, outcome), main findings, and other qualitative notes. Once an agreement was reached, each team member extracted data from a proportion of the remaining included articles (approximately 25%). Throughout the process, the team met regularly to discuss any issues arisen.

We analyzed the data both quantitatively (i.e., numerical descriptive) and qualitatively (i.e., brief summary). We analyzed and reported the articles based on the original labels the authors used. For example, if the authors used the label, “non-technical skills,” we categorized the article under the theme “non-technical skills.” If the label was unclear, we made a team consensus based on what we thought the article best represented. This resulted in four major themes: teamwork and communication, non-technical skills, flow disruption, and teaching or surgical education.

## Results

### Search results

**Fig. 1:**
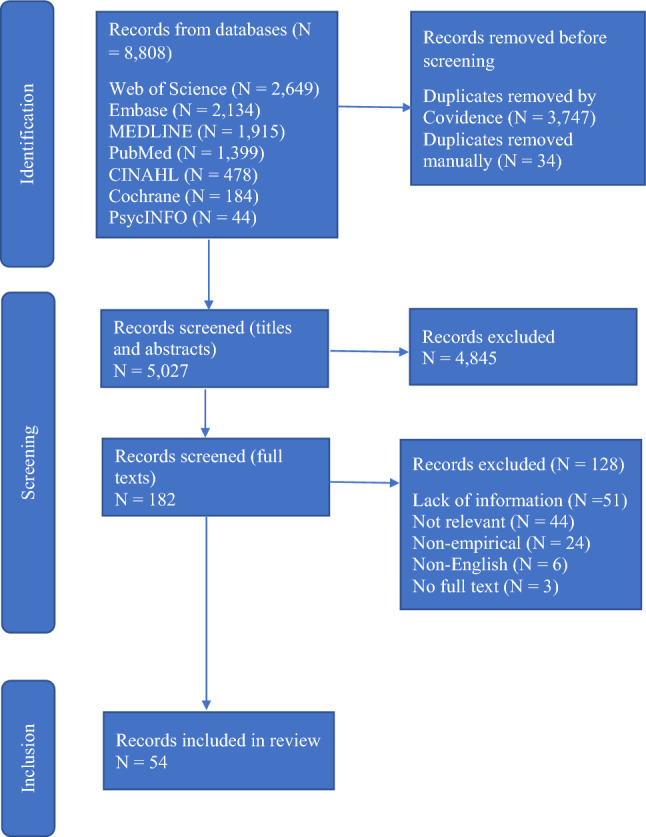
Preferred reporting items for systematic reviews and meta-analyses extension for scoping reviews (PRISMA-ScR) flow diagram

In our initial search, we identified 8808 results and imported them to Covidence. After removing duplicates, 5027 studies were eligible for abstract and title screening. Then, we selected 182 studies for full-text screening. Finally, we included a total of 54 articles in this review. See Fig. 1 for the PRISMA-ScR flow diagram of the screening and selection process.

### Descriptive summary

**Fig. 2:**
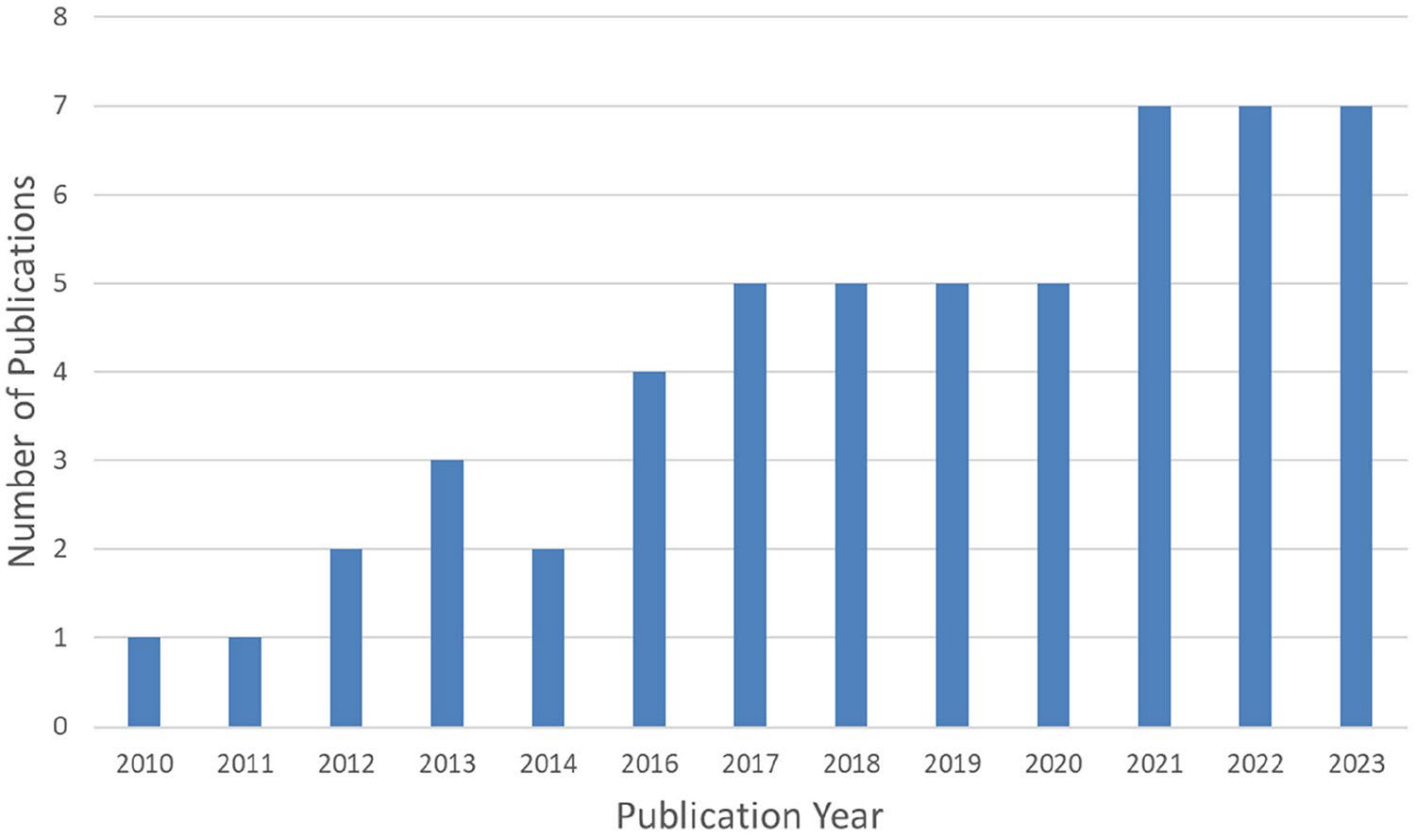
Number of publications by year

We found that articles were published between 2010 and 2023, with a notable increase developing after 2016 (Fig. 2). The majority of studies (*N* = 48, 88.89%) were full-text documents. The articles appeared in diverse types of journals, with a notable skew towards journals in surgical (*N* = 19, 35.19%) and medical (*N* = 14, 25.93%) fields. Surgical specialties in our review followed a distinct distribution, with urology being the most common (*N* = 20, 37.04%), followed by studies incorporating multiple specialties (*N* = 13, 24.07%). Regarding the RAS system used, the da Vinci Robotic System (Intuitive Surgical, California, USA) was the most common (*N* = 27, 50.00%), with the remaining studies not specifying the system. The studies coded were primarily quantitative in nature, using correlational or non-experimental (*N* = 16, 29.63%), quasi-experimental designs or qualitative designs (*N* = 9, 16.67%), and mixed designs (*N* = 8, 14.81%). Most of the studies also leveraged multiple data sources (*N* = 25, 46.30%) or used solely direct observation (*N* = 19, 35.19%) to gather data. Most of the studies’ setting was in or focused on interactions in the operating theater (*N* = 38, 70.37%). The majority of the studies were interested in interactions in the entire operating theater team (*N* = 29, 53.70%) or a subset of the team (*N* = 15, 27.77%).

### Teamwork and communication

Twenty-nine articles examined what was either labeled by the authors as communication or teamwork. The term communication was used very frequently but referred to many different specific variables such as delays of certain lengths [14], the communication of information, such as images, through a system [15], or the subjective rating of quality of communication [16]. Teamwork was used as a broader, more general term that encompassed the basic idea of working together well, or specifically measured specific dimensions associated with teamwork (e.g., communication, cooperation, coordination, leadership, and motivation) that included communication [17]. Some studies did not examine the antecedents and outcomes of effective communication or teamwork in RAS settings but instead described various aspects of communication in terms of who did what [18, 19] or who moved where [20]. Some studies examined how training related to teamwork in RAS settings, with one study demonstrating that a novel algorithm and simulation training improved teamwork as measured by Observational Teamwork Assessment for Surgeons (OTAS) scores improving over 6 months [17] and another using the Delphi technique to develop guidance and protocols for the use of telepresence in remote robotic surgery training [21].

Regarding the outcomes associated with communication and teamwork in RAS settings, many articles discussed how RAS altered the context of teamwork in the operating theater as compared to more traditional surgery, and thus required more explicit multimodal (i.e., verbal and gesture) communication to overcome the challenges posed by the physical configuration and task loads [22–28]. Some other studies found that technological advancements such as spatial communication aids and wireless communication systems improved the quality of communication [16, 29–31]. Finally, similarly to what is known regarding communication in all surgical settings, one study demonstrated that poor communication resulted in worse surgical outcomes in RAS settings [32].

### Non-technical skills

Fourteen empirical articles examined what were labeled as non-technical skills (NTS) in RAS settings. NTS most often referred to various dimensions of team interactions that reflected the non-technical aspects of RAS, including but not limited to teamwork, communication, leadership, decision-making, and situation awareness. These NTS were typically measured using a multidimensional rating scale such as the Interpersonal and Cognitive Assessment for Robotic Surgery (ICARS) rating system [33], the Oxford Non-Technical Skills (NOTECHS) behavioral marker system [34], or the Non-Technical Skills for Surgeons (NOTSS) behavioural rating system [35]. Some of these measurement approaches included self-ratings of NTS, but most utilized expert raters who observed the teamwork and then made judgments regarding the display of various dimensions of NTS during surgery.

The research examining NTS in RAS settings generally focused on three different topics: examining the subjective importance of NTS from the viewpoint of the team members, examining antecedents and outcomes of NTS within RAS, and validating various measures of NTS in RAS settings. Regarding subjective importance, research suggested that team members subjectively endorsed NTS such as communicating effectively, understanding the functions and responsibilities of different team members, and maintaining situation awareness as very important [34, 36]. Regarding antecedents and outcomes of NTS, research demonstrated that NTS improved teamwork effectiveness and other outcomes, including reduced near misses [37–39]. Furthermore, training and other NTS-focused interventions were subjectively appreciated by team members [40] and could increase scores on NTS [33, 41]. One study also found that communicating proactively about risk was associated with higher NTS scores in RAS teams [42]. Finally, a handful of studies were specifically focused on validating NTS measures such as the NOTECHS system [43], the NOTSS system [44], or the ICARS rating system [45]. One study moved beyond such validated scales to demonstrate that sensor-based metrics such as communication, speech, and proximity could be used to measure NTS in the operating theater [46].

### Flow disruption

Seven empirical articles focused on flow disruptions in RAS. Flow disruptions, generally defined as interruptions in the natural surgical workflow, were consistently reported as frequent and negatively impacted outcomes across studies. Common themes identified across the studies included disruptions related to coordination, communication, equipment issues, and patient positioning. However, explicit definitions for these categories were not uniformly provided, and disruptions were typically categorized based on the individual study’s observational schema rather than using a pre-established measure or providing clear operationalized definitions for each category.

Coordination and communication challenges within the operating theater team were frequently cited as significant sources of disruptions, with multiple studies specifically identifying the robot docking and port placement phases as particularly susceptible to delays in coordinating team activities or ensuring that necessary instruments were available [47–50]. Equipment-related disruptions were another consistently identified challenge. Catchpole et al. detailed specific examples of technical complexity, such as incidents where “a curved needle snagged on the port and, owing to a lack of haptic feedback, broke off into the body cavity, requiring an elongated search” [51], [p 3759]. They further explained how robotic technology created new demands and distractions, emphasizing that the reduced direct contact between team members required enhanced communication protocols and checklists to mitigate the issues. Weber et al. similarly observed severe disruptions due to equipment, noting that such disruptions led to the greatest increases in mental workload and situational stress among surgical staff [52].

The impacts of flow disruptions were broadly consistent across the reviewed literature. Al-Hakim specifically detailed disruptions resulting in increasing operative times by over 32% [53]. Koch et al. noted indirect negative effects, such as increased staff workload from frequent equipment and patient-related disruptions, despite not finding direct correlations with patient complication rates [54]. Weigl et al. identified a link between frequent severe disruptions and diminished perceptions of teamwork only among surgeons, emphasizing how these disruptions could undermine team dynamics and the quality of intraoperative teamwork [49].

### Teaching or surgical education

RAS presents unique challenges to interactions between surgical trainers and trainees, such as the remote configuration of the consoles away from the patient where only one person (either the trainer or the trainee) typically has control of the console, lack of non-verbal cues (e.g., gestures), lack of haptic feedback, and lack of joint tissue manipulation and retraction. As a result, surgical trainers need to adapt their teaching by viewing the trainees’ physical movements and positions of robotic arms on the screen and verbalizing their instructions based on visual cues. Surgical trainees also need to adapt their learning by viewing the trainers’ movements on the screen and replicating them based on verbal instructions.

One study developed a taxonomy of teaching behaviors in RAS and identified four behaviors as highly frequent and unique to RAS: providing verbal directions, explaining the trainer’s thought process and decision making, giving the trainee positive reinforcement, and getting the trainee’s head out of the console and engaging in a face-to-face teaching conversation [55]. Another study developed a classification system for providing intraoperative feedback during RAS cases, including types of triggers (e.g., incorrect behavior), feedback (e.g., anatomical), and responses (e.g., asking for clarification), and tested its reliability and generalizability [56]. One conference abstract found that providing feedback on the outcomes of the trainee’s performance reduced task completion time and error rates compared with providing feedback on the task process or no feedback [57]. One study implemented preoperative and postoperative educational discussions (e.g., technical goal setting and feedback giving using Global Evaluative Assessment of Robotic Skills (GEARS)) and found that such discussions reduced discrepancies in trainers’ and trainees’ educational perceptions and improved trainees’ technical performance rated by their trainers [58]. Please see Table 2 for an overview of both quantitative results and qualitative summary.

**Table 2:** Overview of results

Section	# of Articles	% of Total	Results summary
Descriptive summary	54	100.00%	Sharp rise of articles after 2016 during 2010–2023 period Articles published primarily in surgical and medical journals Urology, most commonly studied specialty Da Vinci system most frequently reported Correlational or non-experimental design predominantly used Multiple data sources most commonly used, followed by direct observation Mostly focus on interactions in the operating theater setting Most commonly interested in entire operating theater team's interactions
Teamwork and communication	29	53.70%	Communication studied broadly but specifications were inconsistent (delays, information transfer, subjective ratings) Teamwork assessed as general construct or dimensions (communication, coordination, leadership, motivation) RAS requires explicit multimodal communication (verbal and gesture) Training interventions (algorithm and simulation training) improved teamwork Technical interventions (spatial aids, wireless systems) enhanced communication Poor communication linked to worse surgical outcomes
Non-technical skills (NTS)	14	25.93%	Common measurement systems: ICARS, NOTECHS, NOTSS, expert ratings Three main research foci: subjective importance of NTS, antecedents and outcomes of NTS, validation of measurement systems NTS linked to better teamwork and fewer near misses Training/interventions improved NTS scores Proactive communication about risk associated with higher NTS scores
Flow disruption	7	12.96%	Flow disruptions were frequent Sources of flow disruption: coordination, communication, equipment, patient positioning Varied definitions and categories of sources of disruption Most prone to disruption during robot docking and port placement phases Disruptions increased workload, stress, and operative times Negative impacts on perceived teamwork
Teaching or surgical education	4	7.40%	Frequent teaching behaviors: verbal direction, explaining thought process, reinforcement, pulling trainee for face-to-face teaching Outcome-focused feedback improved task completion time and error rates Pre/post-operative discussions (goal setting and debriefing) improved technical performance and aligned trainer-trainee perceptions

*RAS:* Robotic-assisted surgery; *NTS:* Non-technical skills; *ICARS:* Interpersonal and cognitive assessment for robotic surgery; *NOTECHS:* Oxford non-technical skills; *NOTSS:* Non-technical skills for surgeons; *GEARS:* Global evaluative assessment of robotic skills

## Discussion

The present scoping review sets out to describe how scholars have conceptualized and measured team interactions in RAS. Across 54 included empirical studies, we found that research clustered around four focal themes: teamwork and communication, NTS, flow disruptions, and teaching or surgical education. We also found that standardized terminology and consistent measurement surrounding them remained elusive, particularly for teamwork and communication. This heterogeneity of terminology inhibits cumulative knowledge building; for example, findings about communication span from communication clarity via speakers to subjective ratings of team cohesion, making direct comparison difficult. Simultaneously, clearer progress is evident with NTS, where standardized instruments (e.g., ICARS, NOTECHS, NOTSS) have been applied, and flow disruption, where taxonomies have begun to link flow disruption with team and operative outcomes. Below, we further discuss how these themes manifest in the literature, their implications, and suggestions for future research (See Table 3).

**Table 3:** Summary of suggested future research

Research gap	Suggested future research
Definitional confusion	Be specific about the concepts studied and the terms used and provide their definitions Systematic mapping of team-related concepts in terms of conceptual overlap and distinctiveness
Lack of mediation and moderation	Study team-related concepts as mediators (e.g., different effects of two RAS systems on teamwork which then leads to different outcomes) Study team-related concepts as moderators (e.g., how experience in RAS and teamwork interact to influence outcomes)
RAS teaching and learning	Effective and ineffective teaching techniques or discourse in RAS teaching and learning Training and evaluation on communication, teamwork, or non-technical skills

*RAS:* Robotic-assisted surgery

### Definitional confusion

One of the overarching themes that was noted across topics within this review was a tendency for researchers to use team-related concepts and terms in very exchangeable, loosely defined ways. For example, the term, "communication," was used in many different studies, but referred to many different actual constructs including the occurrence or frequency of specific communication behaviors such as providing verbal direction versus pointing/showing on the console [28], the perceived audio clarity of communication coming through speakers [31], or the perceived general effectiveness of communication within a team [16]. The term, "teamwork," sometimes referred to a multidimensional concept including many facets such as communication, coordination, leadership, and motivation [17] and other times it was conceptualized as an overarching unidimensional concept and measured with a single question such as “how do you rate the teamwork during the procedure?” [52]. This tendency to use the same term to refer to many different concepts makes it difficult to aggregate research findings, as research on the clarity of sound transmitted over speakers is quite different from research examining the perceived effectiveness of information sharing across members of a team.

Exceptions to this pattern were seen in the research on NTS and flow disruptions. This term, "NTS," seems to be more consistently used to refer to the same multi-dimensional set of skills that reflect how well the operating theater team is doing in terms of team behaviors. Research on flow disruptions also seems to use that term in a coherent and consistent manner. In general, however, we suggest that future research on team interactions in RAS settings would benefit from an emphasis on clarity and continuity in term usage. We suggest that future researchers be specific about the concepts they study and the terms they use. For example, if they use the term, “non-technical skills,” as a higher-level construct, we suggest that they study all dimensions of non-technical skills (teamwork, communication, leadership, decision-making, and situation awareness) and provide the definitions. If they study only communication, we suggest that they use the term, “communication.” Furthermore, the research on team interactions in RAS settings may benefit from a systematic “mapping” of team-related concepts in terms of conceptual overlap and distinctiveness.

### Lack of mediation and moderation

One of the gaps in the literature in terms of how team interactions have been conceptualized and conducted is that none of the included articles considered mediators (i.e., explanatory causal mechanisms) or moderators (i.e., variables that interact with one another to determine an outcome). They either conceptualized the team-related concept as an independent predictor of key outcomes such as operative duration, or looked at teamwork as the outcome variable and focused on what predicted effective teamwork. While examining teamwork and team communication as either a predictor or an outcome is certainly an important and useful research question, we suggest that future research on team interactions in RAS settings would benefit from a more theoretically nuanced approach that examines team-related concepts as mediators or moderators.

### RAS training and teaching

This review shows that many efforts have been made to validate various measures of NTS, and there is a recognized importance of team interactions in RAS. However, there is little report on training on NTS or teamwork in RAS or the application of these validated measures in RAS curriculum or training settings. Moreover, due to the definitional confusion, there is a gap in knowledge on what encompasses effective team practices and pitfalls. Furthermore, in RAS, trainers need to verbalize their instructions and actions they want trainees to take, and trainees also need to verbalize what they experience or plan to perform, as only one person typically has the console control. However, there is little research on what is considered effective and ineffective teaching techniques or discourse in RAS teaching and learning. Therefore, we suggest that future research should identify best practices and pitfalls of team interactions not only among the entire RAS team members but also between trainers and trainees in a RAS teaching environment. We also suggest incorporating such knowledge into RAS training and evaluation.

## Limitations

This scoping review has a few limitations. Our literature search was performed in early 2024, and we did not include studies after that time period. Therefore, it is possible that additional studies might be published and missed in this review. We found that more than half of the included articles examined team interactions in the da Vinci Robotic system that most surgeons are very familiar with, and the rest did not specify which system was used in the studies. Recently, newer RAS systems such as Hugo™ RAS System (Medtronic, Minneapolis, USA) and Versius (CMR Surgical, Cambridge, United Kingdom) have been approved for clinical use, and institutions are integrating more than one system into surgical practice [59]. As these newer systems have different designs and interfaces which may require different strategies of team interactions, our results may not generalize to the newer systems. Finally, because of the definitional confusion, there was some difficulty in coding the data, and we made some judgment calls on which one of the four themes the articles primarily reflected. This issue was resolved in the team meetings where the entire research team discussed the articles and reached a consensus.

## Conclusion

Due to the physical configuration of the team in the operating theater, RAS increases the importance of team interactions in effectively and efficiently delivering the surgery. This scoping review summarizes how team interactions have been studied in the literature through four themes: teamwork and communication, NTS, flow disruption, and teaching or surgical education. It also points out the inconsistency in using labels or terminologies for various team-related concepts, identifies gaps in our knowledge, and suggests future research. We hope that this scoping review provides a starting point for future synthesis of evidence (e.g., systematic review, meta-analysis) as well as inspiration for future research to further advance the knowledge regarding team interactions in RAS.

## Supplementary Information

Below is the link to the electronic supplementary material.

Supplementary file1 (DOCX 532 KB)

Supplementary file2 (DOCX 13 KB)

## Data Availability

The data charting form and/or data coded from individual articles will be available upon request and if appropriate (e.g., ethics approved).
