# Supplementary material for: Team interactions in robotic-assisted surgery: a scoping review
Source: J Robot Surg. 2025 Oct 21;19(1):703. doi: 10.1007/s11701-025-02850-z (PMC12540599; doi:10.1007/s11701-025-02850-z)
Supplement: Supplementary file 2 — Supplementary file2 (DOCX 13 KB) [file 11701_2025_2850_MOESM2_ESM.docx]

Supplemental Material 2

Search Terms and Search Query Used in PubMed

**Search Terms**

| robotic surg* OR “robotic surgery” OR “robot-assisted surgery” OR “robot assisted surgery” OR “robotic assisted surgery” OR “robotic-assisted surgery” OR robotic surgical procedure* OR robotic surg* team* OR “minimally invasive surgery” | AND | “non-technical skills” OR “non technical skills” OR “nontechnical skills” OR communicat* OR coordinat* OR cooperat* OR collaborat* OR teamwork OR team process* OR interpersonal skill* OR “information sharing” OR non-technical skill* OR team dynamic* |
| --- | --- | --- |

**Search Query**

**S#1: Title/Abstract**

robotic surg* OR “robotic surgery” OR “robot-assisted surgery” OR “robot assisted surgery” OR “robotic assisted surgery” OR “robotic-assisted surgery” OR robotic surgical procedure* OR robotic surg* team* OR “minimally invasive surgery”

**S#2: MeSH Terms or Subject Terms**

robotic surgery

**S#3**

S#1 OR S#2

**S#4: Title/Abstract**

“non-technical skills” OR “non technical skills” OR “nontechnical skills” OR communicat* OR coordinat* OR cooperat* OR collaborat* OR teamwork OR team process* OR interpersonal skill* OR “information sharing” OR non-technical skill* OR team dynamic*

**S#5: MeSH Terms or Subject Terms**

communication

**S#6**

S#4 OR S#5

**S#7**

S#3 AND S#6
